# Predicting DNA-Binding Specificities of Eukaryotic Transcription Factors

**DOI:** 10.1371/journal.pone.0013876

**Published:** 2010-11-30

**Authors:** Adrian Schröder, Johannes Eichner, Jochen Supper, Jonas Eichner, Dierk Wanke, Carsten Henneges, Andreas Zell

**Affiliations:** 1 Center for Bioinformatics Tübingen (ZBIT), University of Tübingen, Tübingen, Germany; 2 Center for Plant Molecular Biology (ZMBP), University of Tübingen, Tübingen, Germany; Wellcome Trust Sanger Institute, United Kingdom

## Abstract

Today, annotated amino acid sequences of more and more transcription factors (TFs) are readily available. Quantitative information about their DNA-binding specificities, however, are hard to obtain. Position frequency matrices (PFMs), the most widely used models to represent binding specificities, are experimentally characterized only for a small fraction of all TFs. Even for some of the most intensively studied eukaryotic organisms (i.e., human, rat and mouse), roughly one-sixth of all proteins with annotated DNA-binding domain have been characterized experimentally. Here, we present a new method based on support vector regression for predicting quantitative DNA-binding specificities of TFs in different eukaryotic species. This approach estimates a quantitative measure for the PFM similarity of two proteins, based on various features derived from their protein sequences. The method is trained and tested on a dataset containing 1 239 TFs with known DNA-binding specificity, and used to predict specific DNA target motifs for 645 TFs with high accuracy.

## Introduction

As of March 2010, the genomes of 178 eukaryotes were completely sequenced and for another 404 eukaryotic species sequencing projects were in progress [1]. In a large effort, these datasets are annotated by biologists or computational methods [2], [3], and meanwhile they have become one of the most comprehensive resources in biological sciences. By means of the annotation of their domains proteins can be assigned to certain molecular functions (e.g., transcription factor), however, quantitative functional information (e.g., DNA-binding specificities) remain scarce. Despite recent progress in the development of high-throughput technologies for the measurement of protein-DNA interaction parameters as proposed by Maerkl and Quake *et al.* [4] and microarray based technologies for the analysis of TF binding specificities [5], [6], the determination of highly resolved quantitative binding specificity information remains laborious.

Accordingly, comprehensive binding specificity data is only available for a fraction of all known proteins. Only for approximately 3% of all TFs in *Arabidopsis thaliana*, for instance, DNA-binding specificities have been experimentally determined so far. Even for the most intensively studied organisms, i.e., human, mouse and rat, roughly one-sixth of all proteins with annotated DNA-binding domain have been characterized experimentally (see Figure S1). This leads to an enormous gap between the amount of annotated protein sequences and the amount of quantitative binding data.

In the field of qualitative protein function prediction, annotations that were assigned to one protein are often transferred to other proteins with high sequence similarity [7], [8], based on the assumption that similar protein sequences imply similar protein function [9]–[11]. Previously, some approaches were presented that automatically perform such transfers of functional annotations based on sequence similarities [12], [13]. Several similar approaches proceed by extracting the 
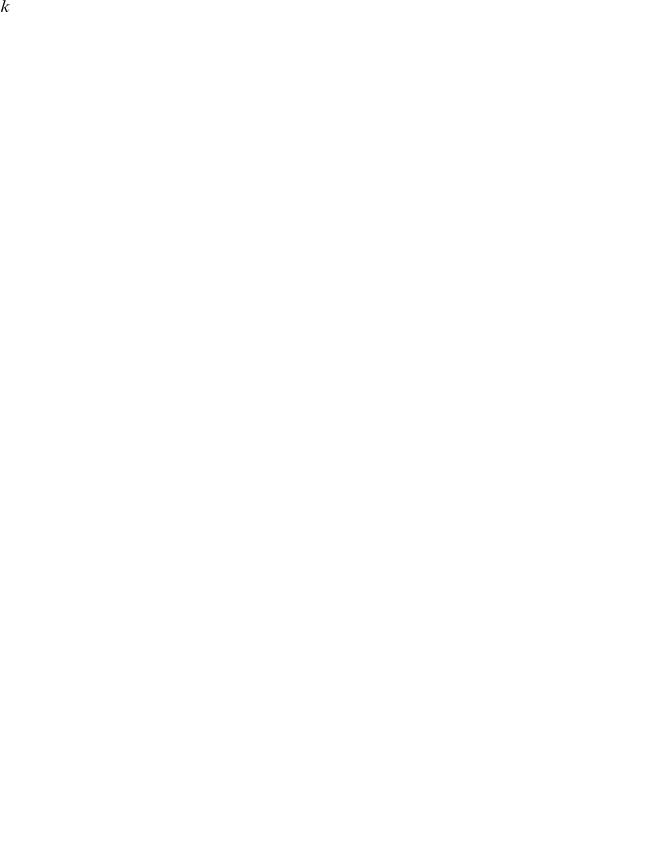
-nearest neighbors for a query protein and then transfer all or the most frequent functional annotations – such as GO terms [14]–[17]. Engelhardt *et al.* used the evolutionary history of proteins as represented by a phylogenetic tree to perform protein function transfers [18]. Brunak *et al.* applied modern machine learning techniques such as artificial neural networks or support vector machines to predict protein annotations based on various features derived from annotated amino acid sequences [19], [20].

Applied to TFs these functional annotations may indicate what structural superclass a certain TF belongs to, for instance ‘*zinc finger*’ [21]. Such annotations, however, do not provide quantitative information, like the DNA-binding specificity of a certain TF, because binding-specificities within TF superclasses, and even within TF classes, may vary tremendously. During the past years, significant progress has been made in our understanding of the biophysical mechanisms underlying the specific DNA-recognition by TFs [22]–[24]. Recently, accurate mechanistic models have been developed to predict physical interactions between TFs and DNA molecules [25], [26]. However, for genome-wide applications, i.e., the computational inference of transcriptional regulatory networks, more simple representations of DNA-binding specificities, such as position frequency matrices (PFMs) are used more commonly [27]–[29]. PFMs indicate for a certain TF how frequent the nucleotides A, C, G, and T occur at each position within the binding site [30]. Thus, to transfer or even predict this type of quantitative information a new approach is needed, which allows to perform transfers of quantitative information with low error rate. To this end, Alleyne *et al.* applied various machine learning methods in order to predict binding profiles of mouse homeodomain TFs [31]. More recently, Alamanova *et al.* proposed a new approach to calculate position weight matrices from protein-DNA complex structures [32]. Toward the challenge of developing a general approach for the prediction of DNA-binding specificities from protein sequences, several questions should be addressed. (1) Which sequence based score is a good quantitative indicator for binding similarity? (2) How large is the error when transferring and recombining quantitative information between proteins? (3) Can this process be automated on large sets of transcription factors? In this work, we developed a method that transfers and combines PFMs between proteins, while addressing each of the open questions. First, instead of using a single pairwise alignment score, we align two proteins with respect to different evolutionary, structural and physicochemical properties. Given these alignments we apply support vector regression (SVR) to infer a quantitative measure for the PFM similarity of two proteins that is based on their protein sequences. This approach is mathematically referred to as distance metric learning, a relatively young discipline in the field of supervised machine learning [33], and has previously not been applied to predict PFM similarities. Based on the SVR model, a framework is implemented that allows to transfer and predict quantitative binding specificity data between TFs. Second, to estimate the average error 5-fold cross-validations with 10 runs is accomplished during the training and the final results are evaluated on a separate dataset that is used for testing purposes only. Third, to show that this method is applicable in large scale we use it to transfer DNA-binding specificity data between TFs to enrich the as yet incomplete annotation of DNA binding consensus motifs of TFs.

## Results

### Functional and sequence datasets

**Figure 1:**
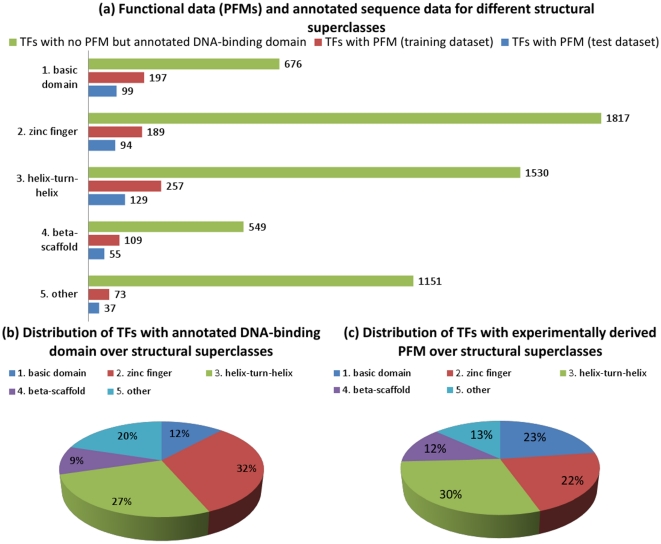
Classification of TFs in structural superclasses. (a) All TFs with known PFM are partitioned with respect to their structural superclass [34]. The number of PFMs are given by the non-redundant dataset compiled in this work (Section ‘DNA-binding specificity databases’), which is split into a training and a test dataset. (b) Distribution of TFs with annotated DNA-binding domain over structural superclasses, (c) Distribution of TFs with experimentally derived PFM over structural superclasses.

To train our approach a sufficient number of TFs has to be collected for which quantitative binding specificity information, the protein sequence and the DNA-binding domain annotation is available. We collected binding specificity data (PFMs) from several databases, such as TRANSFAC® (see Table 1), and protein sequences with annotated DNA-binding domains from sequence databases, such as UniProt (see Section ‘Protein sequences, DNA-binding domain annotations and TF-classifications’) focusing on eukaryotic species. These data was retrieved and merged into a non-redundant dataset that contains 1 239 eukaryotic TFs with known PFM (see Section ‘DNA-binding specificity databases’). We partitioned this dataset according to the five structural superclasses of TFs [34], following the assumption that TFs from different superclasses bind distinct DNA-motifs and should therefore be treated independently. The five structural superclasses are: (1) basic domain (*basic domain*), (2) zinc-coordinating DNA-binding domains (*zinc finger*), (3) helix-turn-helix (*helix-turn-helix*), (4) beta-scaffold factors with minor groove contacts (*beta-scaffold*) and (5) other transcription factors (*others*). In addition, each superclass specific dataset was subdivided into a training and a test set with a ratio of 2∶1 (see Figure 1(a)). Then, a second dataset was compiled that contains proteins for which no PFM but the protein sequence and the DNA-binding domain annotation could be retrieved. This dataset contains 5 723 TFs that were also partitioned according to their structural superclass. In a later stage of this work, PFMs from proteins in the first dataset were combined and transferred to proteins in the second dataset, while estimating the average error. The classification of TFs with annotated DNA-binding domain and/or PFM to the five structural superclasses is shown in Figure 1(a) and compared to the estimated number of existing TFs [35]. It becomes obvious from Figure 1(a) that the number of TFs without PFM is by far larger than the number of TFs with experimentally determined PFM. Figure 1(c) shows the distribution of all TFs with known PFM over the structural superclasses. The largest number of PFMs was obtained for the *helix-turn-helix* class and the lowest number of PFMs was obtained for the class *others* even though *others* is the second largest superclass (see Figure 1(b)). In the Figure S1 the number of experimentally derived versus predicted PFMs among six of the most intensively studied model organisms including *Homo sapiens*, *Mus musculus*, *Rattus norvegicus*, *Arabidopsis thaliana*, *Drosophila melanogaster* and *Saccharomyces cervisiae* is depicted.

**Table 1:** Databases that provide models of DNA-binding specificities.

database	covered species	models	reference	URL
TRANSFAC®	eukaryotes	846	[64]	http://www.biobase-international.com/
JASPAR core	multicellular	123	[30]	http://jaspar.cgb.ki.se/
YEASTRACT	*S. cerevisiae*	284	[53]	http://www.yeastract.com/
SCPD	*S. cerevisiae*	23	[65]	http://rulai.cshl.edu/SCPD/
AGRIS	*A. thaliana*	65	[66]	http://arabidopsis.med.ohio-state.edu/
FlyReg	*D. melanogaster*	184	[67]	http://www.flyreg.org/

The shown databases cover different organisms and contain varying numbers of models that are stored in different formats (PWMs, IUPAC motifs, or PFMs).

### Predicting PFM similarity from annotated protein sequences

**Figure 2:**
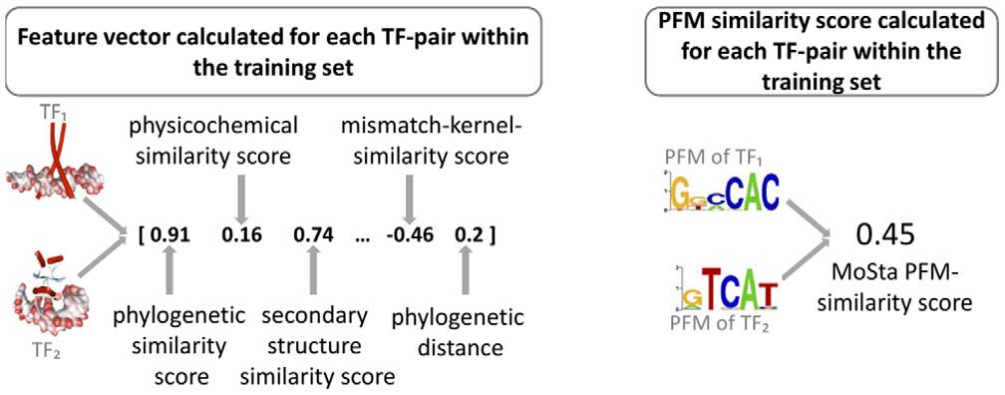
Training of SVR model to predict PFM similarities. An SVR-based supervised machine learning approach is used to predict pairwise PFM similarities based on various features, derived from amino acid sequences of the DNA-binding domains of pairs of TFs. To this end, for each TF pair in the training set, a feature vector consisting of phylogenetic, physicochemical and structural domain similarity scores is computed. All pairwise PFM similarities in the training set are quantified using MoSta [36]. Next, a support vector machine is trained to predict PFM similarities based on the sequence-derived feature vectors. In machine learning, this methodology is referred to as supervised distance metric learning [33].

**Figure 3:**
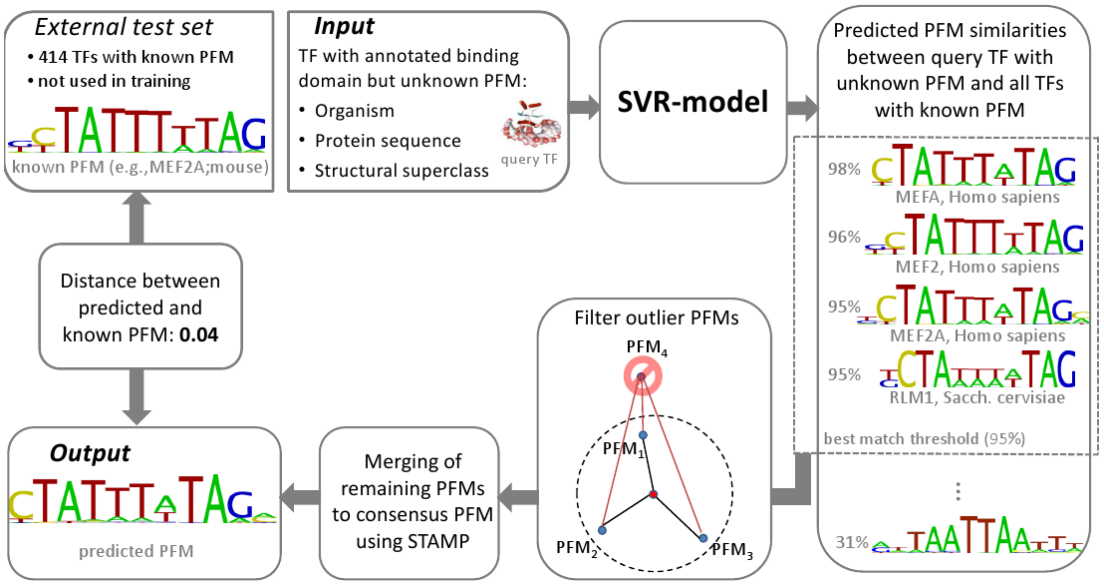
PFM prediction framework and error estimation. The prediction framework takes the following information about the query TF as input: (1) the corresponding organism, (2) the entire protein sequence, (3) the interval spanned by the DNA-binding domain, (4) the structural superclass of the TF. The query TF is compared to all TFs with known PFM and identical superclass. Pairwise feature vectors are computed and the similarity between the known PFMs and the unknown one of the query TF are predicted based on SVR. Next, the best matches, i.e., the TFs for which a PFM similarity above a predefined threshold (default: 0.95) was predicted, are merged to a consensus PFM using STAMP [37]. To assure that the merged PFMs are sufficienly similar, an outlier filter is applied before merging in order to remove dissimilar PFMs causing inhomogeneity of the best matches. In the shown example, four best matches were found for MEF2A, a mouse beta scaffold myocyte enhancer factor with known PFM taken from the test set. The best matches are mostly MEF isoforms from human with known PFMs. Since these PFMs are very similar to each other, no outliers have to be removed before merging. The predicted consensus PFM was compared to an experimentally detemined PFM in order to assess the error in terms of normalized MoSta units [36]. The error between the predicted and the annotated PFM is 0.04, which precisely agrees with the average PFM similarity of the best matches (0.96) predicted by the SVR model.

The presented approach can be partitioned into two stages. The first stage comprises the training of SVR models, i.e., one model for each of the five structural superclasses, that quantitatively predict the functional similarity (i.e., PFM similarity) of TFs based on sequence homology and other features derived from their annotated protein sequences (see Figure 2). In the second stage these SVR models are used to transfer PFMs to TFs of interest (see Figure 3).

#### First stage: Training of SVR model to predict PFM similarities

In the first stage a training set of TFs with known PFMs and annotated protein sequences is used to learn PFM-similarities based on support vector regression. To this end, for each pair of TFs in the training set a vector of 30 different pairwise similarity scores in compiled with respect to various evolutionary, structural and physicochemical properties. Most of those pairwise similarity features are derived from the amino acid sequences of the annotated DNA-binding domains. A comprehensive list of all pairwise similarity scores can be found in Table 2. The results of these pairwise comparisons are used to train an SVR model that predicts PFM-similarities which are quantified using the well established multiple alignment based PFM similarity score MoSta [36]. In machine learning, this process, i.e., the learning of similarities/distances from various features, is referred to as distance metric learning [33]. Figure 2 depicts the training of this supervised machine learning approach. For each pair of TFs in the trainings set, feature vectors consisting of all 30 similarity features are compiled. Based on these vectors, which represent the binding domain similarities, the SVM is trained to learn the PFM similarities.

**Table 2:** Similarity score calculation methods and their parameters.

Similarity type	substitution matrix	parameter	reference
**Alignment of the DNA-binding domains [Needleman-Wunsch ** [**68**] **]**			
1. Sequence identity	BLOSUM62	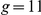 , 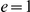	[69]
2. Sequence similarity	BLOSUM62	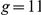 , 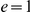 , 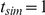	[69]
3. Sequence similarity	BLOSUM62	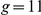 , 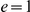 , 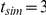	[69]
4. Sequence similarity	BLOSUM62	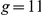 , 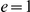 , 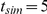	[69]
5. BLOSUM based	BLOSUM62	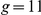 , 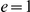	[69]
6. PAM based	PAM80	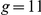 , 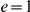	[70]
7. PAM based	PAM10	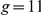 , 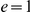	[70]
8. Secondary structure based	LUTR910102	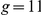 , 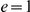	[71]
9. Secondary structure based	MEHP950101	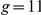 , 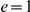	[72]
10. Secondary structure based	MEHP950102	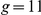 , 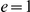	[72]
11. Secondary structure based	MEHP950103	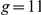 , 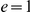	[72]
12. AA-contact frequencies based	MIYS930101	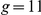 , 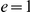	[73]
13. AA-pair distance based	MIYT790101	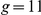 , 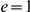	[74]
14. Structure based	NIEK910102	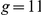 , 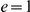	[75]
15. Structurally related proteins based	RISJ880101	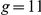 , 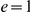	[76]
16. Physical feature based	WEIL970101	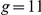 , 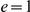	[77]
**Alignment of the DNA-binding domains [Local Alignment Kernel (LAK) ** [**58**] **]**			
17. BLOSUM based	BLOSUM62	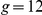 , 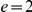 , 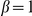	[69]
18. BLO based	BLO62	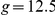 , 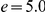 , 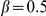	[78]
19. PAM based	PAM250	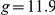 , 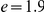 , 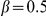	[78]
20. LAK optimized	GCB	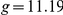 , 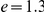 , 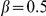	[78]
21. LAK optimized	JTT	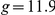 , 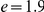 , 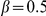	[78]
**Alignment of the DNA-binding domains [MisMatch Kernel (MMK) ** [**59**] **]**			
22. Number of matching subsequences	–	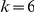 , 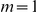	–
23. Number of matching subsequences	–	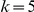 , 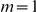	–
24. Number of matching subsequences	–	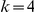 , 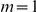	–
**Alignment of the DNA-binding domains [SVM-pairwise ** [**60**] **]**			
25. SVM-based	BLOSUM62	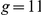 , 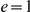	[69]
26. SVM-based	PAM80	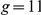 , 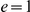	[70]
**Alignment of the flanking regions of the DNA-binding domains [Needleman-Wunsch ** [**68**] **]**			
27. BLOSUM based	BLOSUM62	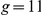 , 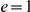 , 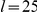	[69]
28. BLOSUM based	BLOSUM62	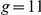 , 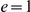 , 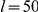	[69]
**Alignment of the predicted secondary structures of the whole proteins [Needleman-Wunsch ** [**68**] **]**			
29. Similarity of predicted secondary structure	custom build	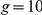 , 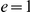	–
**Phylogenetic distance of the species of two proteins**			
30. Phylogenetic distance	–	–	[79]

For each feature the method, its parameters and, when needed, the substitution matrix are provided. The parameters 
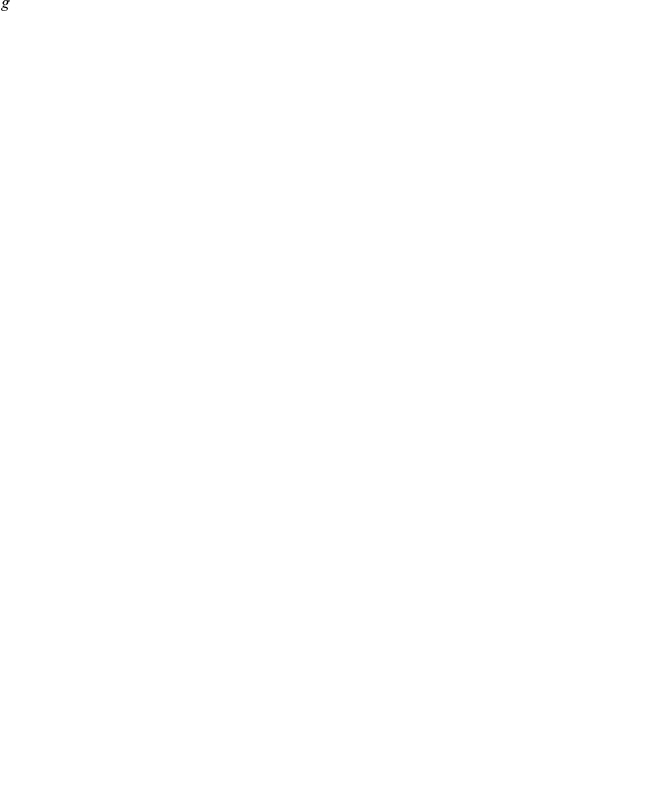
 and 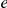 give the gap opening and gap extension penalties and 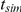 gives a similarity distance threshold below which two amino acids are still considered a match. The parameters 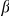 and 
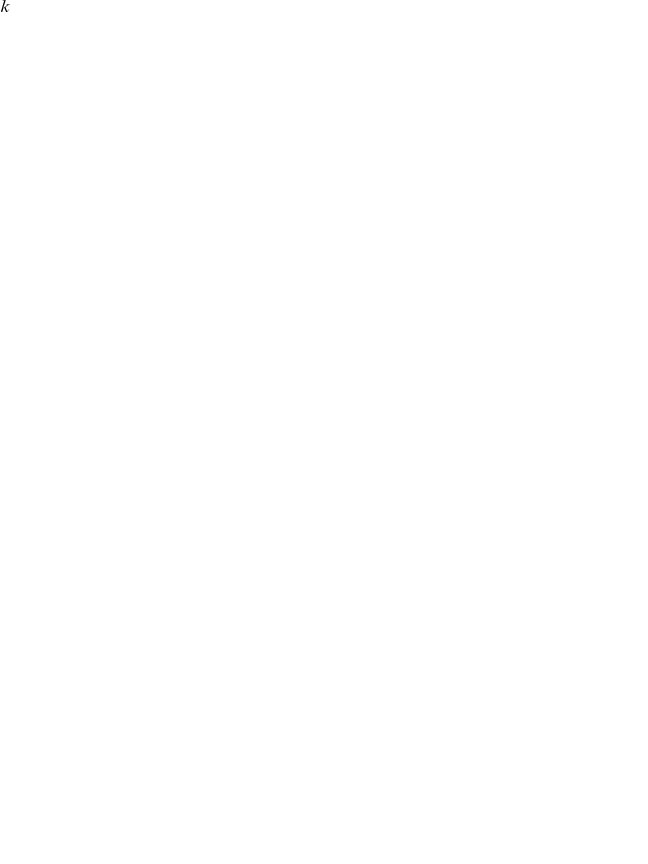
, 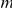 are parameters of the local alignment and mismatch kernel, respectively. The parameter 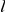 defines the length of the flanking regions considered for the alignment.

#### Second stage: PFM prediction and error estimation

In this stage PFMs are combined and transfered to query proteins that either lack PFMs or that are used for testing purposes (see Figure 1). The prediction framework requires for any given query TF three pieces of information. First, the respective organism of the query TF, which is required to derive the phylogenetic feature. Second, the sequence of the annotated DNA-binding domain, from which most of the remaining features are derived. Third, the structural superclass of the DNA-binding domain, because one model for each structural superclass was trained individually. Given this information, pairwise similarities between the query TF and all other TFs with known PFM are predicted using the corresponding SVR model of the respective structural superclass. The best matching PFMs, if any, are further processed an merged to a consensus PFM in the remaining steps. First, outliers are detected and removed. Second, the consensus PFM is generated using the STAMP PFM merging algorithm which is described elsewhere [37]. The resulting consensus PFM is finally returned as output for the respective query TF. It is important to note, that not for any given query TF an output is generated. If no similarities to known PFMs are predicted by the SVR models, no PFM prediction can be performed.

In order to estimate the prediction error, an external test set is compiled consisting of 414 TFs with known PFMs which are not used in the SVR-training procedure (see Figure 1). The failure between predicted and original PFM is quantified in terms of MoSta units [36]. A detailed example for a myocyte enhancer factor (MEF-2A), a mouse beta scaffold TF available retrieved from TRANSFAC public, is depicted in Figure 3. Three MEF isoforms from human are predicted by the respective beta scaffold SVR model to be highly similar to the PFM of the query factor and all lie above the required best match threshold of 0.95. These PFMs are very similar to each other such that no outliers need to be removed. The error between the predicted consensus PFM and the real PFM is 0.04. Thus, the estimated PFM similarity of 0.96 precisely agrees with the observed error.

The overall error rate is estimated for each structural superclass individually by calculating the average absolute error (AAE), i.e., the average [0,1]-normalized distance between predicted and annotated PFMs in terms of MoSta units [36]. The reader is referred to the Methods section, for a formal description of the AAE (see ‘Validation of the SVR models and predicted PFMs’).

### Sequence based PFM similarity measure

#### Results of the most predictive SVR models for each structural superclass

**Figure 4:**
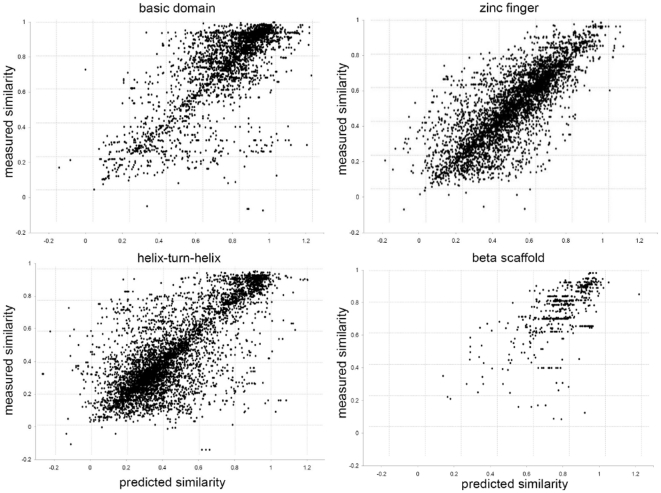
Predicted versus experimental similarities for different structural superclasses. Each dot indicates the predicted and known PFM similarity of all TF-pairs in the test dataset. The 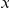-axis gives the sequence based SVR-based PFM similarity prediction and the 
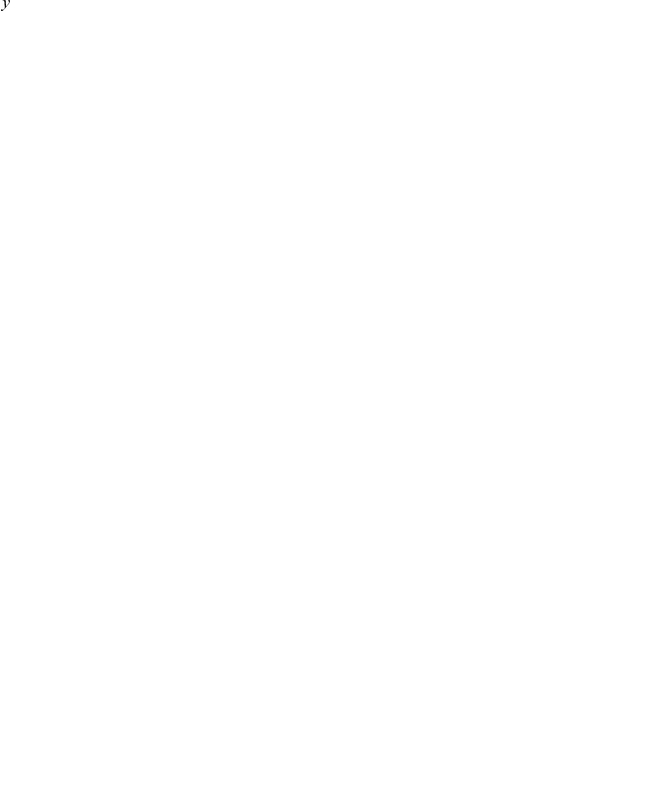
-axis gives the similarity of their known PFMs. Thereby, for each structural superclass the best SVR model from the training dataset was used for the predictions. The Pearson correlation-coefficients are as follows: basic domain: 0.77, zinc finger: 0.80, helix-turn-helix: 0.77 and beta-scaffold: 0.64.

For each structural superclass one SVR model was derived from the training datasets that contain binding specificity data (PFMs). Thereby, the objective of every SVR was to learn a quantitative relationship between the sequence based features (see Table 2) and the PFM similarity of the TFs (see Section ‘Low-level similarity score for PFMs’). To obtain robust estimates of the predictive performance of the SVR models for each structural superclass 5-fold cross-validations with 10 runs of repeated random partitionings was performed. During this process feature selection was dismissed, as it did not have a positive impact on the prediction performance. Thus, all SVR models were trained with all features. Finally, for each structural superclass the predictive performance of the derived SVR models was evaluated on the test dataset. The results of these tests indicate a linear relationship between the predicted and the measured PFM similarity scores (see Figure 4), where the Pearson correlation coefficients are: *basic domain*: 0.77, *zinc finger*: 0.80, *helix-turn-helix*: 0.77, *beta-scaffold*: 0.64 and *others*: 0.69. The respective average absolute errors (AAEs) on the test datasets are: *basic domain*: 0.093, *zinc finger*: 0.087, *helix-turn-helix*: 0.098, *beta-scaffold*: 0.080 and *others* 0.137. The errors on the test and training set are in a similar range, which indicates that the models have a good ability to generalize. For the class *others* the AAE on the training set (0.095) was lower than on the test set (0.137), thus the generalization ability of this model is not optimal, which may be due to the small number of training points and the structural diversity of the contained TFs (see Figure 1).

#### Analysis of individual pairwise similarity measures

**Figure 5:**
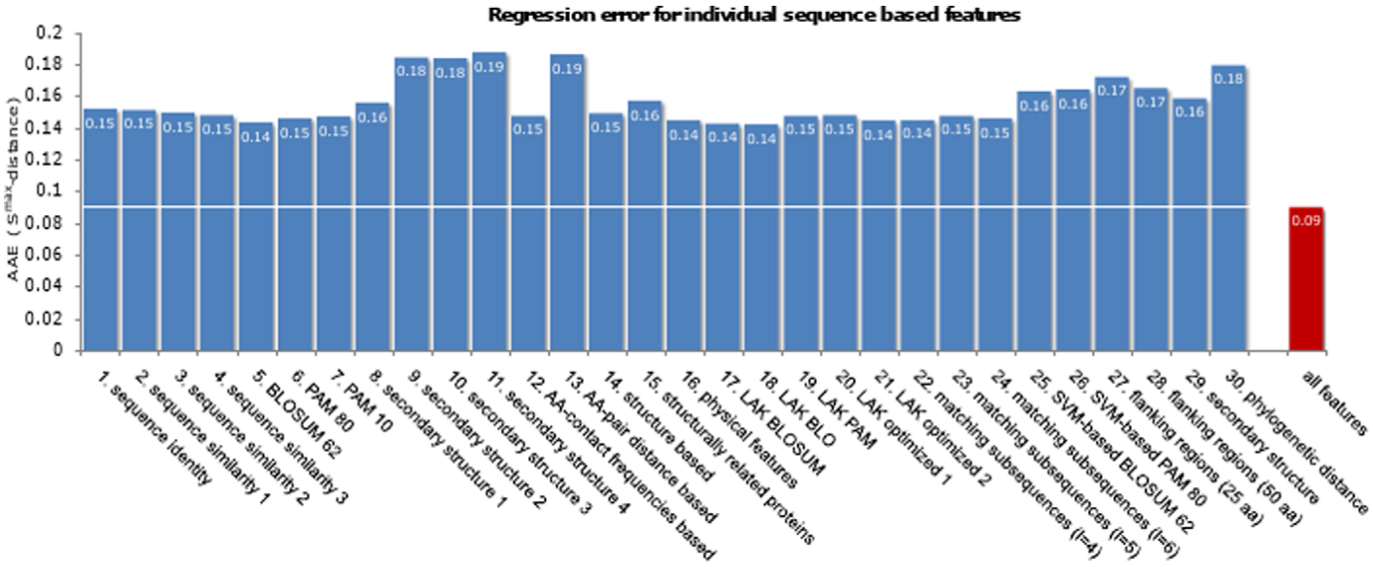
Regression error when using individual sequence based features. Depicted are the average regression errors when training the SVR with a single feature for the superclass *zinc finger*. These error estimations are performed with a 10×5 cross-validation on the trainings dataset. The ‘all features’ bar indicates the average regression error when training on all 30 features. This evaluation is performed to assess the prediction performance of SVR models trained on single features individually compared to the prediction performance of SVR models trained on all features. These results suggest that the 30 feature SVR performs best to learn linear relationships between DBD- and PFM-similarities.

**Figure 6:**
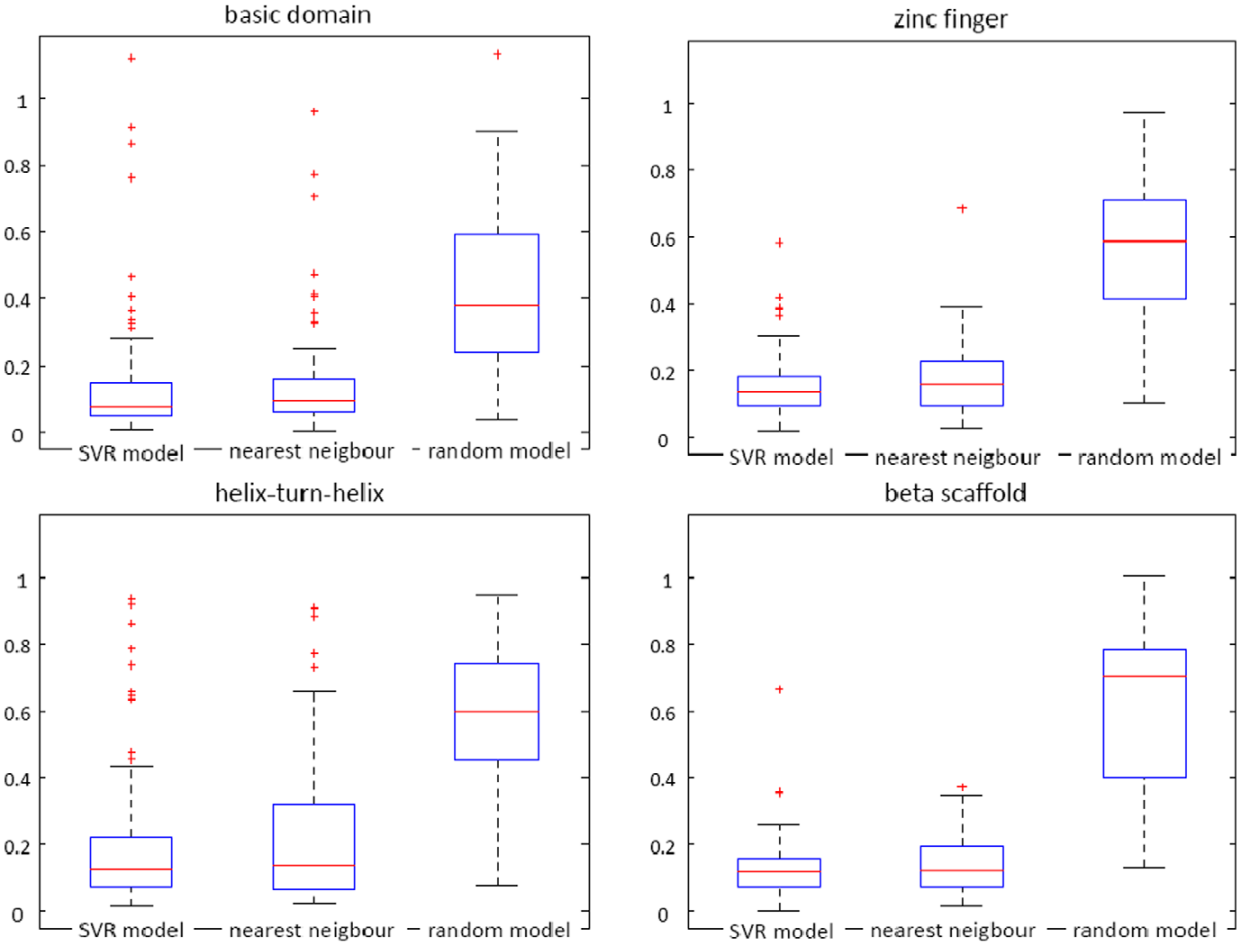
PFM transfer error of the SVR-based method compared to nearest neighbor algorithm and a random model. The box plots show the distribution of the AAE, i.e., the mean distance between predicted and annotated PFMs in terms of normalized MoSta units [36], when applying the SVR model, the nearest neighbor, and a random model to the test set. The errors are calculated separately for the structural superclasses 1–4. The average error of the SVR model is in all four structural superclasses slightly lower than the average error of nearest neighbor algorithm and the random model (see median and 75th percentile).

In the previous section the results of PFM similarity predictions based on 30 sequence based features were shown. Here, the question arises if an individual feature is sufficient to infer PFM similarity and accordingly, how much is the benefit of combining the 30 features using support vector regression? To determine if individual features are already sufficient to quantitatively predict PFM similarity, an SVR was trained and tested separately for each of the 30 features on the structural superclass with the highest Pearson correlation coefficient, namely *zinc finger*. The AAE for each individual feature is given in Figure 5. This analysis shows that the AAE increases about 60% when comparing the SVR trained on 30 features against the best SVR that was trained on an individual feature. These results suggest that the combination of 30 different sequence-derived features performs best to learn linear relationships between sequence- and PFM-similarities. We additionally assessed the prediction performance for diverse subsets of the 30 features, selected based on PCA [38] and RankProp [39], respectively (data not shown). Based on the observation that the PFMs predicted by the all-feature classifier performed best, we concluded that every individual feature contributes to the overall prediction performance. Note that this evalutation does not assess PFM transfer errors (these are shown in Figure 6), but regression errors of SVR models.

### Transferring PFMs between TFs

#### Prediction of PFMs for TFs with known PFM

After deriving SVR models to predict PFM similarities, these models were used to transfer PFMs to TFs without PFMs (see Figure 3). Before applying this procedure, however, the average error of such PFM-transfers was estimated on the test dataset that contained 413 TFs with known PFM. The results of this analysis are depicted in Figure 6, along with the AAEs of a random model (Section ‘Prediction framework based on a random model’) and a nearest neighbor algorithm (Section ‘Prediction framework based on nearest neighbor algorithm’), which was additionally implemented in this work. The AAEs of the framework with the default parameters averaged over all TF classes is 0.12 on a scale from 0 to 2. In comparison, the average similarity 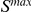 (see Materials and Methods for details) between two PFMs that are randomly sampled from the same structural superclass is 0.64, indicating that the predicted performance of the SVR model is significantly higher than the performance expected by random guessing. Moreover, we observed that the average PFM similarity between two PFMs, which are associated with the same TF and result from different wet lab experiments, is approximately 0.1 in terms of normalized MoSta units [36]. Thus, against the background of this experimental variance, the SVR-based method hits the limits of what is possible with respect to the prediction accuracy. The SVR based approach yields slightly lower error rates in all structural superclasses (see median and 75 percentile in Figure 6). On average, however, also the nearest neighbor approach yields satisfying low errors. These outcomes confirm the findings of Alleyne *et al.*, who suggested that for mouse homeodomain TFs nearest neighbor algorithm is well suited to predict binding profiles [31]. Our results suggest that this assumption also holds for the general case. The cause of these findings might be that the set of TFs without PFM is dominated by trivial cases, in which PFMs of orthologs from other organisms are available. The nearest neighbor algorithm might benefit for this reason. Examples of non-trivial cases are depicted in Figures S3 and S4. Furthermore, as additionally mentioned in the discussion, similarities learned by the SVR model correlate on the full similarity scope with the true PFM similarity of two PFMs (see Figure 4). Simple sequence similarity features, however, such as the domain similarities of two TFs with respect to the BLOSUM62 substitution matrix on which the nearest neighbor algorithm is base, weakly correlate with the true PFM similarity of two PFMs as depicted in Figure S2. The SVR model should be preferred in applications were besides the best matching TF also lower similarities or even dissimilarities are of interest. In conclusion, the strength of the novel approach proposed in this work is that this method computes a prediction score, which is highly correlated with the true PFM similarity of two TFs, by integrating various weakly correlated sequence similarity measures.

#### Prediction of PFMs for TFs with unknown PFM

After estimating the AAE on the test sets, PFMs of TFs with previously unknown PFMs are predicted. Therefore, all 5 723 TFs without known PFM are used as input for the prediction framework (see Figure 3 and File S3). Please keep in mind that a transfer is only performed for query TFs that have a predicted PFM similarity to TFs with known PFM of at least 95% in terms of normalized mosta units [36]. With these settings the PFMs of 645 TFs were transferred. These TFs are distributed among the structural superclasses as follows: 166 *basic domain* (26.5%), 180 *zinc finger* (28.7%), 207 *helix-turn-helix* (33%), and 73 *beta-scaffold* (11.6%), where the percentage indicates the fraction of query TFs for which a reliable prediction could be made. This corresponds to an average transfer rate of 11.3% for any given query TF. All TFs along with their transferred PFMs are available in the File S1.

#### Examples of transferred PFMs

**Figure 7:**
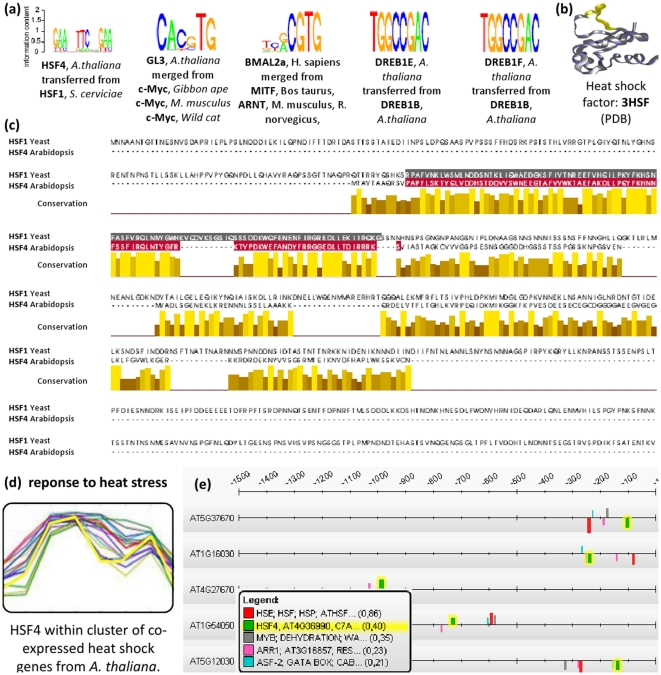
Examples of PFMs transferred to different TFs. (a) Depicted are five examples of PFMs that are transferred to the query TF. These transfers merge PFMs from different species and the final PFM is depicted as sequence logo. (b) Depicts the physical structure of the DNA-binding domain of HSF4 from *A. thaliana* [61] that is drawn with BallView [62]. (c) For HSF4, the query and the best matching TF are aligned with JalView and their DNA-binding domains are colored [63]. This alignment contains a gap consisting of eleven amino acids in the DNA-binding domain of the HSF1 from *S. cerevisiae*. The amino acids that constitute the gap in the alignment are drawn yellow within the physical structure (see (b)). From this structure it can be seen that the colored amino acids do not affect the canonical *helix-turn-helix* structure of the HSF that is responsible for specific DNA-binding [40]. (d) Depicts *A. thaliana* cluster of co-expressed genes that contains HSF4 and 15 other heat shock genes, which was derived with EDISA [42]. (e) Depicts promoter scans of these genes; several matches of the predicted HSF4 PFM were detected by ModuleMaster [29].

PFM prediction examples for several TFs with unknown DNA-binding specificity are shown in Figure 7 (a). Besides two examples of trivial PFM transfers between DREB1 variants in *A. thaliana* two examples are given, where similar PFMs from different species are merged to consensus PFMs and transferred to the query TFs from *H. sapiens* and *A. thaliana*. One further example from this figure is HSF4 from *A. thaliana* which was predicted to have a similar binding specificity as HSF1 from *S. cerevisiae*. Thus, the respective PFM was transferred from HSF1 to HSF4. To visualize the DNA-binding domain similarity their aligned protein sequences are depicted in Figure 7 (c). This alignment shows that the HSF1 from *S. cerevisiae* contains eleven amino acids in the DNA-binding domain that cannot be aligned against the DNA-binding domain of HSF4 from *A. thaliana*. By analyzing the structure of the HSF1 DNA-binding domain, one can see that these amino acids are not contained in the canonical *helix-turn-helix* structure of HSF1 [40], and may therefore leave the DNA-binding specificity unaffected (see Figure 7 (b)). Thus, despite differences at the protein sequence level, HSF4 and HSF1 are strongly conserved at the DNA-binding domain level and are therefore likely to bind to similar regulatory sequences on the DNA [9]. In order to check this hypothesis, the transferred PFM for HSF4 is used to scan a set of co-expressed heat shock genes from *A. thaliana* for significantly enriched transcription factor binding sites (TFBSs). The heat shock gene cluster was obtained by clustering stress-response microarray data conducted by Kilian *et al.* [41]. In this work, Kilian *et al.* exposed *A. thaliana* shoot and root cells to heat and other stress conditions and conducted time-series to measure the transcriptional response. A set of 16 genes was found to be co-expressed under different heat stress conditions using EDISA [42] (see see Figure 7 (d)). Among these, 10 genes were found to be known heat shock genes by gene set enrichment analysis (corrected p-value 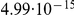). Next, the promoter sequences of these genes were scanned for *cis*-regulatory modules using the ModuleMaster algorithm [29]. As explained in more detail in the Methods section (see ‘Application to sets of co-expressed genes’), ModuleMaster uses a multi-objective optimization approach to find TFBS enrichments in clusters of co-expressed genes. ModuleMaster found matches of the transferred PFM of HSF4 significantly enriched in the heat shock cluster, indicating a regulatory relationship between HSF4 and the heat shock genes, which is also confirmed by literature [43]. As additional source of evidence, the expression profile of HSF4 was found to be strongly correlated to the heat shock genes as detected by ModuleMaster (see yellow expression profile highlighted in Figure 7 (d)). The result of the *cis*-regulatory module detection is depicted in Figure 7 (e). Shown are promoter sequences (1500 bp upstream of TSS) of 5 heat shock cluster genes and the *cis*-regulatory module binding sites, respectively. The TFBSs associated with the HSF4 PFM are highlighted in yellow. A set of further non-trivial PFM predictions is depicted in Figures S3 and S4. A comprehensive list of all PFM predictions can be found in File S1.

## Discussion

In this work we presented a new method to transfer quantitative information between proteins, which is based on the assumption that similar DNA-binding domain sequences imply similar transcription factor binding specificities. To apply this method to the problem of transferring DNA-binding specificities between TFs, a comprehensive dataset covering PFMs, DNA-binding domain annotations, protein sequences and structural superclass annotations was compiled. This dataset gave insights into the current availability of DNA-binding specificity data and was the basis for training and evaluating our method. Thereby, several questions were approached: (1) Which sequence based score is a good quantitative indicator of PFM similarity? (2) How large is the quantitative error when transferring quantitative information to another protein? (3) Can this process be automated on large scale problems?

Regarding the first question, we found that the prediction of PFM similarity based on a single pairwise alignment is subject to large errors, when compared against our SVR models that were based on 30 features. The average error of the PFM similarity predictions was below 0.1 (on a scale from 0 to 2) for all structural superclasses, except *others*. Furthermore, we observed a high correlation between known and predicted functional similarities for the structural superclasses 1–4. *Zinc finger*, for instance, had a correlation of 80%. Hence, the prediction of functional similarity should be based on multiple features, at least in case of the given application.

Regarding the second question, the average absolute error of PFM-transfers on the test dataset was 0.12 with an average transfer rate of 11.3%. Thus, the transfers to the 5 723 TFs without PFM had a low coverage but high specificity and reliability. Overall, the presented framework could be used to predict the PFMs of 645 TFs with high accuracy, which are provided in File S1. This constitutes a significant improvement in the number of TFs with known PFMs. Even if the overall coverage remains low the SVR models allow to predict the PFM for any TF, whose annotated protein sequence and structural superclass is known.

In this work, we apply the presented framework to predict DNA-binding specificities to TFs with unknown PFMs. The approach is based on distance metric learning, i.e., we train a model to estimate the similarity of the DNA motifs recognized by two TFs, based on the similarity of their DNA binding domains. By using this model, we are able to identify TFs with known PFM which bind to similar DNA motifs than a particular TF of interest with unknown binding specificity. The PFMs of the TFs for which the highest PFM similarity to the TF of interest was predicted, are in turn merged to generate the predicted PFM. In order to assess how much the PFM prediction benefits from the combination of different sequence derived features through the SVR model, we additionally implemented a nearest neighbor based approach that screens the database of TFs with known PFMs and simply transfers the PFM from the one TF with the most similar binding domain sequence. The results from this comparison suggest on the one hand, that the SVR approach performs in all cases better than the nearest neighbor, but shows on the other hand, that the nearest neighbor approach often yields on average comparable results. It should be kept in mind that on average the similarity between two PFMs, which are associated with the same TF and result from different wet lab experiments, is approximately 0.1 in terms of normalized MoSta units [36]. Thus, on average both methods hit for some structural superclasses (i.e., helix-turn-helix) the limits of what is possible with respect to the prediction accuracy. A second advantage of the prediction framework presented in this work compared to nearest neighbor methods or similar approaches is the accurat similarity measure predicted by our approach, i.e., our method computes a prediction score which is highly correlated with the true PFM similarity of two TFs, by integrating various weakly correlated sequence similarity measures (see Figure 4). Conversely, the predictions performed by the nearest neighbor approach are directly resulting from a single weakly correlated feature, such as the domain similarities of two TFs with respect to the BLOSOM62 substitution matrix. As depicted in Figure S2, linear relationships between sequence similarity and PFM similarity of pairs of TFs only exist in regions above 90% sequence similarity. Similarities learned by the SVR model, however, correlate on the full similarity scope with the true PFM similarities (see Figure 4). Thus, the SVR model should be preferred in applications were not only the best matching TF, but also lower similarities or even dissimilarities are of interest. Furthermore, the SVR model constitutes a means of estimating the true PFM similarity of two TFs, even if their DNA binding profiles are both unknown. It thus serves as a starting point for further analyses, such as the hierarchical clustering of TFs based on the similarity of their PFMs and the computation of probalistic models in order to derive families of PFMs. These can subsequently be used as prior knowledge to increase the detection sensitivity of motif inference algorithms such as SOMBRERO [44], PRIORITY [45] or NestedMICA [46].

## Materials and Methods

### Models and datasets

#### DNA-binding specificity models

PFMs model the DNA-binding specificity of TFs. They store position specific nucleotide frequencies in a matrix 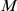 of size 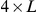, where 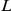 is the length of the binding motif and each row represents one nucleotide. For instance, entry 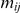 specifies the frequency of nucleotide 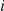 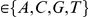 at position 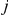 in a multiple alignment of observed binding sites. Other common models are motifs in IUPAC (Union for Pure and Applied Chemistry) code or PWMs (Position Weight Matrices). PWMs are similar to PFMs, but they store the log-likelihood ratios of the nucleotide distributions and are often normalized with respect to background probabilities at each position. IUPAC representations model each position in the binding site through a IUPAC-letter that represents one or more nucleotides (e.g., 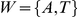).

Here, the standard representation are PFMs. To convert a PWM into a PFM, each entry is normalized by its column's sum, converting the number of occurrences into frequencies. To convert a IUPAC representation into a PFM, a column is constructed by giving all nucleotides of the respective IUPAC letter equal weight, again assigning frequencies to every nucleotide.

#### DNA-binding specificity databases

Several databases exist that contain models of DNA-binding specificities for eukaryotes (e.g., PWMs, IUPAC motifs, or PFMs). An overview of the databases used in this work is given in Table 1. All models contained therein are retrieved and converted into PFMs. Thus, we obtain a list of TFs with one or more PFMs assigned. In TRANSFAC® some PFMs are associated with TF complexes or are marked as familial binding profiles; these entries are removed from the dataset, since these PFMs cannot be linked to one TF. The corresponding data is provided in the File S2, PFMs from TRANSFAC®, however, are removed from this file since they are proprietary.

Whenever multiple PFMs are available for one TF a consensus PFM is generated. For this purpose STAMP is used [37]. Initially, STAMP was developed to generate familial binding profiles (FBPs) for certain classes of TFs. Here, instead, STAMP will be used to integrate multiple PFMs that are associated with one TF. STAMP is applied with an ungapped Smith-Waterman alignment.

#### Protein sequences, DNA-binding domain annotations and TF-classifications

For all TFs in TRANSFAC® the protein sequences, protein domain annotations and the structural classes are retrieved. TFs extracted from other databases are mapped to these TFs through their SwissProt identifier. To restrict protein annotations to DNA-binding domains, each protein domain is mapped to its respective GO-annotation in Pfam and only considered when classified as ‘DNA-binding’. In addition, the structural class of every TF is obtained from TRANSFAC® [34]. From this classification the superclass of every TF is extracted. If for any TF the protein sequence or DNA-binding domain is unavailable, the TF is removed from the dataset.

#### TFs without known PFM

To predict novel PFMs for TFs a dataset containing TFs with unknown PFMs is compiled. In this dataset all TFs are used which have: no PFM, a protein sequence, a DNA-binding domain and a known structural superclass. Protein sequences and DNA-binding domain annotations are taken from UniProt [8].

### Sequence and PFM similarity measures

#### Low-level similarity score for PFMs

To compare two PFMs to each other their similarity (or distance) has to be quantified. Here, the 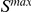, 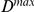 scores are used, which were published in 2008 by Pape *et al.* [36]. In this scoring system, for two PFMs 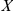 and 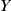, the number of binding site overlaps with an offset 
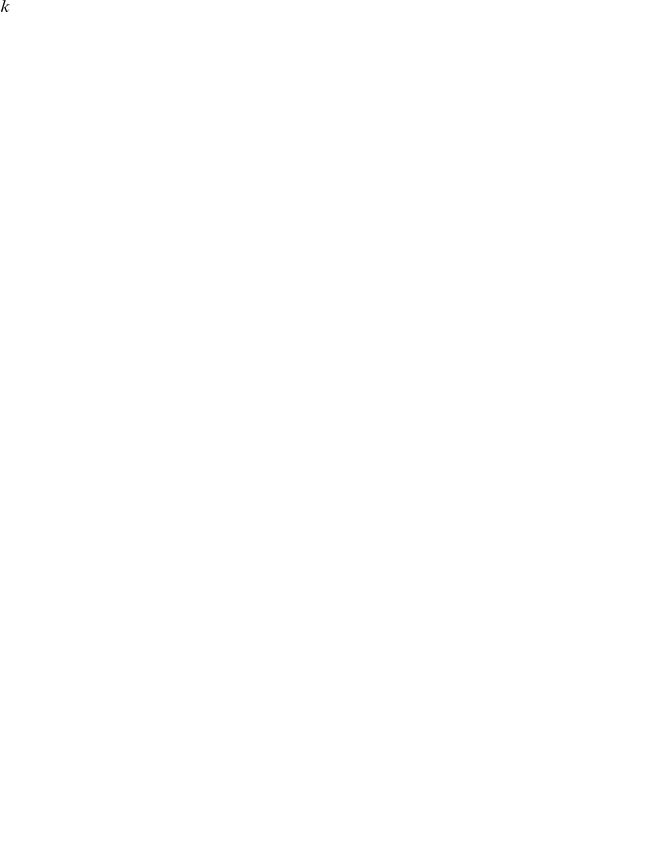
 is determined on a random DNA sequence. This figure is then divided by the product of the individual binding site probabilities (Equation 1).
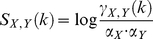
(1)


Thereby, 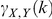 denotes the frequency of 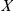 having a binding site that overlaps at the 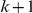-*th* position with a binding site of 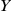. The terms 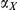 and 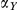 give the probabilies for an occurrence of an binding site for 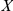 and 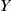 under the background model 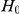. The maximal similarity score 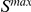 for two PFMs is calculated by considering all possible overlaps 
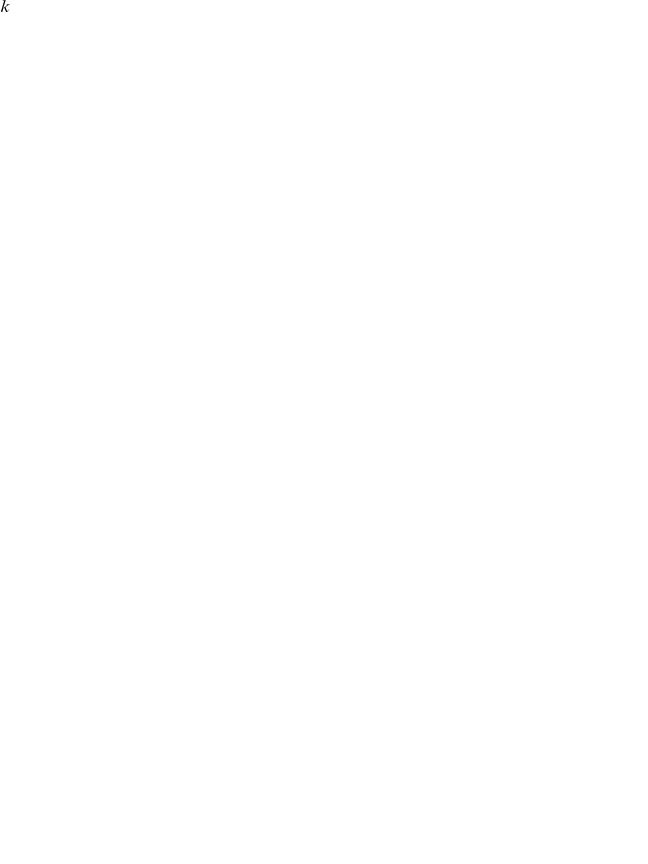
 in combination with the different orientations (sense 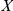 and antisense 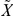) (Equation 2).
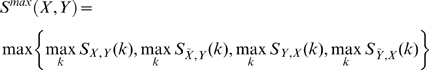
(2)


In some cases it is desirable to calculate the distance of two PFMs, rather than their similarity. To transform the 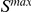 similarity score into a distance measure the following formula is applied:
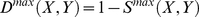
(3)


The 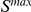, 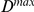 scores can be calculated for every TF pair with known PFMs and thus provide a label for supervised learning.

#### Sequence based similarity scores (features)

To derive a sequence based similarity measure that allows to predict PFM similarities, first several alignment based similarity scores are calculated, which constitute the basis for deriving the final similarity measure. Here, 30 low-level similarity measures (features) are derived that are based on local alignments of DNA-binding domains, flanking regions of DNA-binding domains, the alignment of secondary structure predictions and taxonomic distances. These alignments are performed with different substitution matrices and different alignment methods. As substitution matrices BLOSUM and PAM are used, as well as different physicochemical substitution matrices from the AAindex2 database [47]. As alignment methods Needleman-Wunsch and several kernel methods are used. The mismatch kernel, however, does not explicitly align the sequences. If multiple DNA-binding domains are annotated in one or both TFs all domains are compared to each other and the best similarity score is returned. In addition to these alignment based features the taxonomic distance of TFs is provided, which is taken from NCBI. All methods and parameters used to generate these features are shown in Table 2. Overall, for every pair of TFs a vector 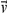 is obtained that has 30 entries (features), each providing a different measure of similarity.

### Structuring and preprocessing the test and training datasets

Given the 30 sequence based low-level similarity scores 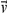 (Table 2) and the label (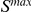), the aim is to learn a model that predicts 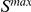 when only provided with sequence based information 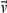. As indicated earlier an SVR is employed to learn the optimal similarity score. This approach is applied separately for each TF superclass, since TFs from different superclasses are not expected to exhibit sufficient structural and functional similarity. Thus, the dataset is split according to the TF superclass annotations taken from TRANSFAC®. In addition, for each TF superclass, one third of all TFs are put aside as test dataset.

Before the SVR is applied several preprocessing steps are performed. First, all features and labels are normalized between -1 and 1, with the following formula:
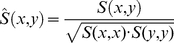
(4)


Thereby, 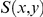 denotes the similarity score calculated by comparing some property of two TFs 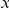 and 
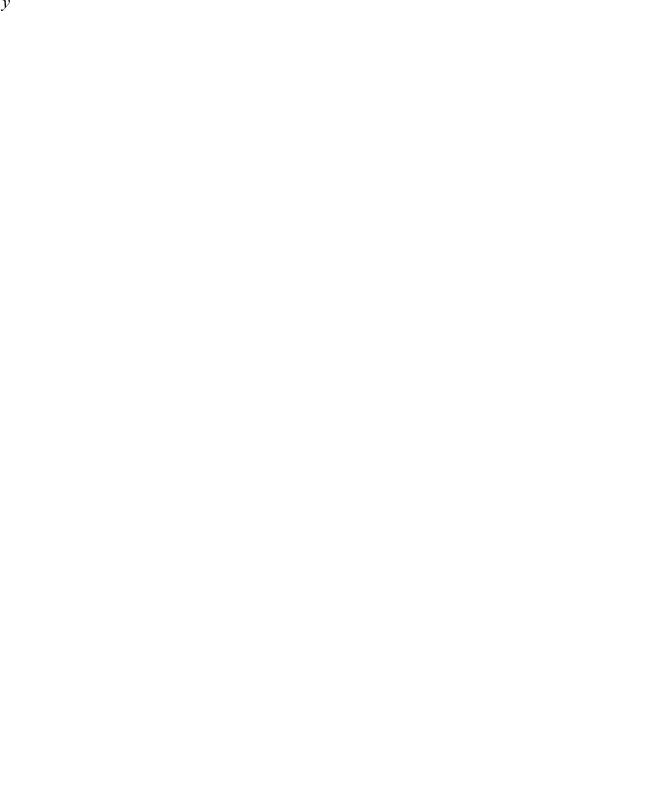
, whereas 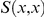 and 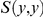 denote the similarity score when comparing the respective TF to itself.

### Learning the PFM similarity score

To learn the PFM similarity score all TF-pairs with a normalized BLOSUM62-score of their DNA-binding domains over 0.3 are considered for training, these are referred to as local TF-pairs (see Figure S2). Furthermore, TFs that have a 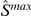 similarity score of one are removed from the training and test set, to avoid learning TFs that have been assigned to the same PFM. For the remaining TF-pairs the similarity vectors 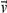 are calculated and combined into a training matrix with 30 columns (features), and one row for each considered TF-pair. For each row the label is calculated by the normalized 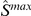 similarity score. Such a training dataset is constructed for each of the five TF superclasses.

#### Support vector regression

To train the SVR model on these datasets cross-validation and parameter optimization are employed. On each training set a 5-fold cross-validation is performed with 10 runs of repeated random partitioning, hence a 10×5-fold cross-validation. This repeated cross-validation is intended to provide a robust regression error even when testing for different SVR parameters. As SVR method, the 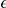-SVR with RBF kernel is used. The SVR parameters 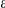 and 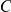, and the RBF-kernel parameter 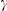 are optimized by a grid search. Thereby, the following parameters are considered 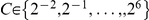, 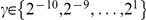 and 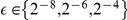. To quantify a regression error for the predictions made by each SVR model the average absolute error (AAE) is calculated, on the test partitions of the cross-validation. After compiling the training set with this procedure for every structural superclass, the SVR is applied to each training set as described above. The final SVR model, for each structural superclass, is the model with the minimal AAE.

### PFM prediction framework

#### Prediction framework based on the trained similarity measure

The trained SVR models do not directly predict PFMs, but the similarity of the DNA-binding specificities of two TFs. To perform a PFM transfer a framework is implemented, which makes use of the trained SVR models. As input the algorithm expects the protein sequence of a query TF, with its annotated DNA-binding domain, the structural superclass and the species. Then the SVR model is used to predict the PFM similarity of the query TF to all TFs with known PFM in the same superclass, whenever their DNA-binding domain similarity exceeds 0.3 (normalized Needleman-Wunsch alignment score with BLOSUM62). This provides a list of TFs with predicted PFM similarities to the query TF. From this list all TFs with a predicted similarity under a certain threshold are removed (default: 0.95). From the remaining list the 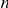 (default: 5) TFs with the highest predicted similarity are kept. If multiple PFMs remain after these filtering steps, an outlier detection is performed. Therefore, for each TF 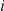, the average 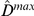-distance 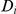 to all other TFs in TF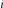 is computed (Equation 5). Moreover, the average 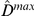-distance 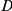 of all TF-pairs is calculated (Equation 6). If for any TF 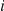 the ratio of its distance to the other TFs divided by the average overall distance (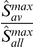) exceeds a certain threshold (default: 1.5) this TF is removed.
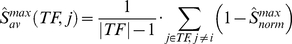
(5)

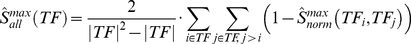
(6)


After removing the outlier PFMs, the remaining PFMs are merged into one FBP (using STAMP). This consensus PFM then constitutes the predicted DNA-binding consensus motif for the query TF. An overview of the framework is given in Figure 3. This framework is applied to all TFs in the dataset that contains TFs without PFMs (Section ‘TFs without known PFM’).

#### Prediction framework based on nearest neighbor algorithm

To compare the prediction accuracy of our SVR-based method against a naive supervised learning approach, we implemented a prediction framework based on the nearest neighbor (NN) algorithm. The algorithm simply transfers the PFM of the TF for which the highest DNA-binding domain similarity to the given query factor was computed. The domain similarities were measured in terms of an alignment score with respect to the BLOSUM62 substitution matrix. As the SVM-based framework requires the existence of a TF with known PFM which has sufficient domain similarity to the given query TF, it did not permit the prediction of a PFM for the entirety of all TFs comprised by the evaluation set. However, to ensure a fair comparison we computed the AAE on the same number of TFs for the SVM-based and the NN-based framework. The included TFs were selected based on the predicted PFM similarity for the SVR method and based on the domain similarity score for the NN algorithm.

#### Prediction framework based on a random model

To compare the SVR models against a random guesser, the prediction framework is implemented with random TF picks instead of the SVR model. This framework proceeds in the same manner as the SVR based framework, however, after determining the number of best matches the corresponding TFs are neglected and resampled from all TFs of the same structural superclass.

### Validation of the SVR models and predicted PFMs

To validate the PFM prediction framework and the similarity scores their results are compared against the test dataset. First, the SVR models are tested for their ability to predict the PFM similarity of local TF-pairs. For this analysis the PFM similarity of all local TF-pairs in the training datasets are predicted with the respective SVR model and compared against the known PFMs. To assess the quality of the predictions the Pearson correlation coefficient 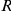 and the AAE are calculated for each structural superclass. The PFM prediction framework is validated by performing a PFM prediction for every TF in the test dataset, and comparing the result against the corresponding annotated PFM by means of the 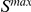 similarity score. The AAE for a respective structural superclass consisting of 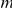 query TFs is calculated as follows (Equation 7)
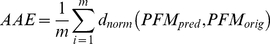
(7)where 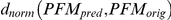 gives the 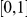-normalized distance between predicted and known PFMs in MoSta units [36].

### Application to sets of co-expressed genes

In subsequent computational analyzes, known and predicted PFMs of different organisms were used to scan clusters of co-expressed genes from microarray data sets for *cis*-regulatory modules (CRMs). CRMs are sets of transcription factor binding sites (TFBSs), which are found in physical proximity on promoter sequences of co-expressed genes and are often used to detect regulatory relationships [48]–[50]. In this work, we use the ModuleMaster algorithm for CRM detection [29].

ModuleMaster retrieves promoter sequences from the Ensembl database [51] for all genes within each cluster of co-expressed genes. Next, the predicted PFMs are converted to PWMs and together with PWMs (see RSA-tools for details [52]) from YEASTRAC [53], JASPAR [54] and TRANSFAC® [34] used to scan through these promoter sequences. Furthermore, binding motifs provided by the PLACE transcription factor binding database were integrated [55]. In order to derive the binding score of a single PWM 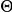 on subsequence 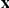 of sequence 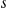, ModuleMaster calculates weight scores, which were first introduced by Aerts *et al.*

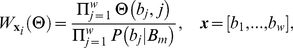
(8)where 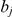 is the nucleotide found at position 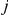 in the subsequence 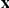, 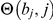 is the probability of finding 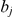 according to PWM 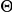 and 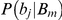 is the probability of finding 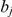 according to the background model 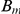. As background model, sequences from a 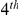-order hidden Markov model that was derived from coding sequences of the respective organism were used. 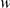 is a parameter that gives the length of the respective binding site. There are three different strategies to calculate appropriate individual cutoff levels, which minimize the amount of false positive and/or false negative hits, as proposed by Kel *et al.*: (1) Minimization of false negatives, (2) Minimization of false positives, (3) Minimization of a combination of both [56]. The second cutoff strategy was used to pre-calculate individual weight score cutoff values for all PWMs. These individual cutoff values are used during matrix scan in order to decide, if a match at a certain position within the promoter sequence should be counted as TFBS or not. After matrix scan, ModuleMaster searches for CRMs using a multi-objective genetic algorithm that takes the weight scores and multi-variate correlations between TFs and target genes on the expression level into account.

### Implementation and availability

The core of the prediction framework, from which various libraries and external programs are called, is implemented in Java. The source code of the prediction framework is licensed under the GPL 3 and available at http://code.google.com/p/pfmprediction/.

The alignments are performed with BioJava [57]. 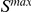 similarity scores are calculated with the program MoSta provided by Pape *et al.* [36].

The PWMs are merged with a local copy of STAMP, obtained from Mahony *et al.* [37]. To train the SVR model LIBSVM was used (available at http://www.csie.ntu.edu.tw/~cjlin/libsvm). A C-implementation of the local alignment kernel was provided by Saigo *et al.* [58]. Leslie *et al.* provided source code for the calculation of the mismatch kernel [59]. An implementation of the SVM pairwise score was obtained from Liao *et al.* [60].

## Supporting Information

Figure S1 Distribution of TFs with and without PFMs for six different species. The absolute numbers of TFs per species are taken from the work of Wilson et al. (www.transcriptionfactor.org) and reflect TFs predicted by HMMs. The number of known PFMs is taken from the integrated dataset compiled in this work (see File S3) and compared to the number of transferred PFMs. The availability of PFMs heavily depends on the species of interest. *S. cerevesiae*, for instance, has the best coverage of TFs with known DNA-binding specificities, whereas for *H. sapiens* the largest number of PFMs are available. Interestingly, the number of newly predicted PFMs is highest for *M. musculus* and *H. sapiens* and worst for *S. cerevisiae*.
(2.25 MB TIF)

Figure S2 Sequence versus PFM similarities for all TF pairs. Depicted are sequence similarities of DNA-binding domains versus PFM similarities for different structural superclasses. To learn the PFM similarity score, all TF pairs with a normalized BLOSUM62-score of their DNA-binding domains over 0.3 are considered for training; these are referred to as local TF-pairs.
(2.84 MB TIF)

Figure S3 Set of non-trivial example predictions. Depicted are several examples of non-trivial PFM transferrers from the test set, for which the prediction error is estimated. The best matches, i.e., the TFs for which a PFM similarity above a predefined threshold (default: 0.95) was predicted, are merged to a consensus PFM using STAMP. The predicted PFM similarity for each best match is given in brackets. Depicted are the sequence logos of the merged consensus PFM. The prediction error in terms of normalized MoSta units quantifies the distance between known and predicted PFMs.
(3.19 MB TIF)

Figure S4 Examples of non-trivial PFM transfers between TFs from distinct classes. This figure shows sequence logos, PFM similarity scores and TF class affiliations where either one (first column) or all best matches (second column) belong to a different TF class than the query TF. We found that for 51 TFs (70%) of the query TFs all of the predicted best matches belong to the same TF class. For the remaining 12 TFs (30%), we observed that at least one of the best matches was from another class than the query TF and for 6 of these 12 TFs (15%) we found that all best matches were from another class. In most of these cases PFMs of TFs of class 1.2. (Helix-loop-helix (bHLH)) were transfered to TFs of class 1.3. (leucine zipper (bHLH-ZIP)) and vice versa.
(0.50 MB TIF)

File S1 Predicted TFs. File S1 contains all 645 TFs for which PFM transfers were performed by our prediction framework. For each TF various annotations are provided, i.e., UniProt ID, species information, protein sequence, DNA-binding domain annotation and the ID of the best matching PFM that was predicted by our method. All PFM models are listed in File S2.
(0.52 MB TXT)

File S2 Binding specificity models. The integrated PFM-dataset containing all PFM models of the test and trainings sets is provided in File S2. For the models from TRANSFAC Professional no matrix is given as they are proprietary.
(0.71 MB TXT)

File S3 All query TFs in input format. File S3 contains all 5723 TFs without experimentally derived PFMs but annotated DNA-binding domains. This dataset was used as input file for the prediction framework presented in this work.
(4.21 MB TXT)
