# Supplementary figures and images for: Predicting DNA-Binding Specificities of Eukaryotic Transcription Factors
Source: PLoS One. 2010 Nov 30;5(11):e13876. doi: 10.1371/journal.pone.0013876 (PMC2994704; doi:10.1371/journal.pone.0013876)

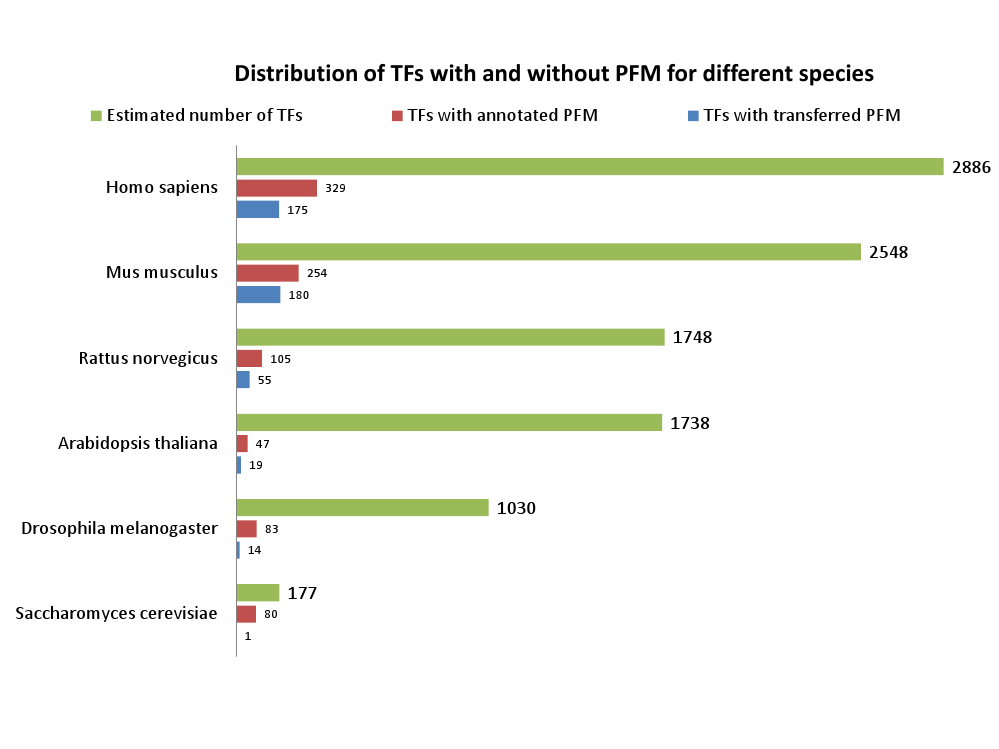

Supplement: Figure S1 — Distribution of TFs with and without PFMs for six different species. The absolute numbers of TFs per species are taken from the work of Wilson et al. (www.transcriptionfactor.org) and reflect TFs predicted by HMMs. The number of known PFMs is taken from the integrated dataset compiled in this work (see File S3) and compared to the number of transferred PFMs. The availability of PFMs heavily depends on the species of interest. S. cerevesiae, for instance, has the best coverage of TFs with known DNA-binding specificities, whereas for H. sapiens the largest number of PFMs are available. Interestingly, the number of newly predicted PFMs is highest for M. musculus and H. sapiens and worst for S. cerevisiae. (2.25 MB TIF) [file pone.0013876.s001.tif]

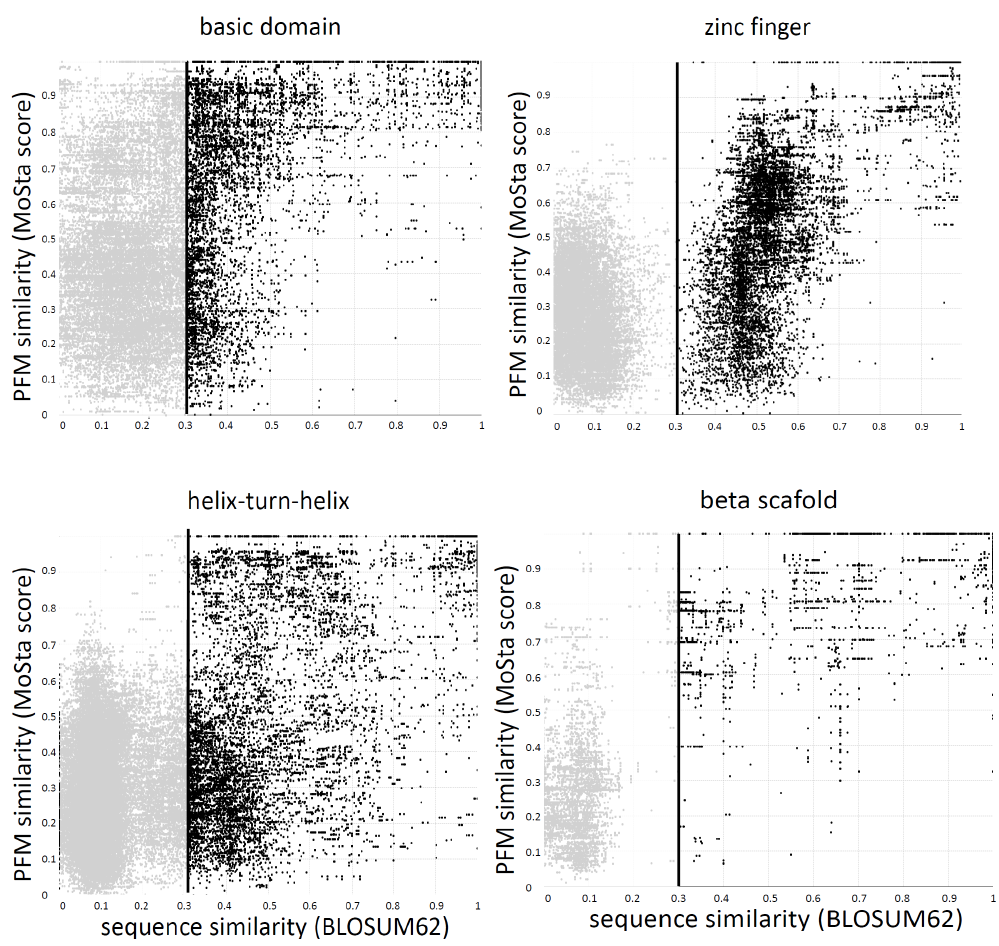

Supplement: Figure S2 — Sequence versus PFM similarities for all TF pairs. Depicted are sequence similarities of DNA-binding domains versus PFM similarities for different structural superclasses. To learn the PFM similarity score, all TF pairs with a normalized BLOSUM62-score of their DNA-binding domains over 0.3 are considered for training; these are referred to as local TF-pairs. (2.84 MB TIF) [file pone.0013876.s002.tif]

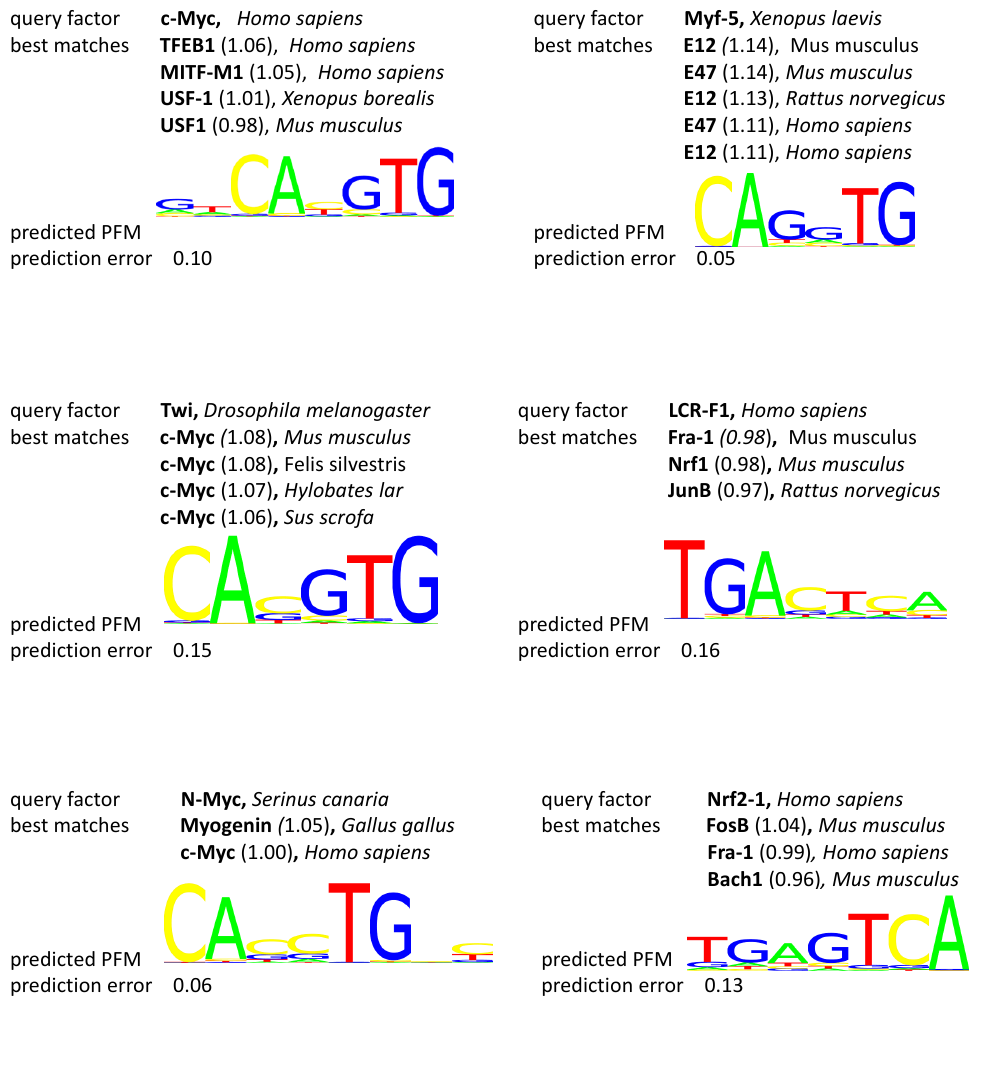

Supplement: Figure S3 — Set of non-trivial example predictions. Depicted are several examples of non-trivial PFM transferrers from the test set, for which the prediction error is estimated. The best matches, i.e., the TFs for which a PFM similarity above a predefined threshold (default: 0.95) was predicted, are merged to a consensus PFM using STAMP. The predicted PFM similarity for each best match is given in brackets. Depicted are the sequence logos of the merged consensus PFM. The prediction error in terms of normalized MoSta units quantifies the distance between known and predicted PFMs. (3.19 MB TIF) [file pone.0013876.s003.tif]

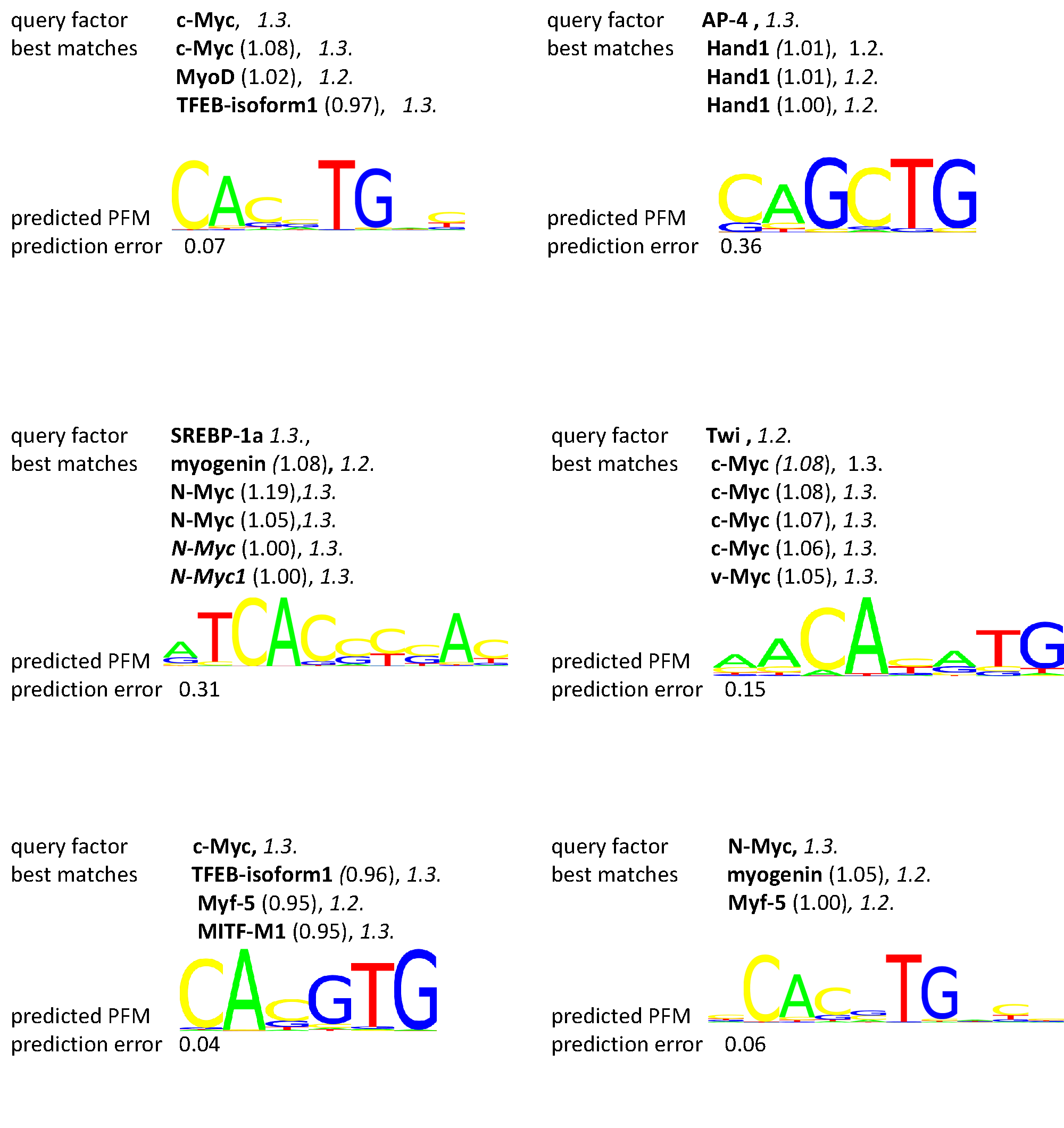

Supplement: Figure S4 — Examples of non-trivial PFM transfers between TFs from distinct classes. This figure shows sequence logos, PFM similarity scores and TF class affiliations where either one (first column) or all best matches (second column) belong to a different TF class than the query TF. We found that for 51 TFs (70%) of the query TFs all of the predicted best matches belong to the same TF class. For the remaining 12 TFs (30%), we observed that at least one of the best matches was from another class than the query TF and for 6 of these 12 TFs (15%) we found that all best matches were from another class. In most of these cases PFMs of TFs of class 1.2. (Helix-loop-helix (bHLH)) were transfered to TFs of class 1.3. (leucine zipper (bHLH-ZIP)) and vice versa. (0.50 MB TIF) [file pone.0013876.s004.tif]
